# Shift in Social Media App Usage During COVID-19 Lockdown and Clinical Anxiety Symptoms: Machine Learning–Based Ecological Momentary Assessment Study

**DOI:** 10.2196/30833

**Published:** 2021-09-15

**Authors:** Jihan Ryu, Emese Sükei, Agnes Norbury, Shelley H Liu, Juan José Campaña-Montes, Enrique Baca-Garcia, Antonio Artés, M Mercedes Perez-Rodriguez

**Affiliations:** 1 Department of Psychiatry Icahn School of Medicine at Mount Sinai New York, NY United States; 2 Department of Signal Theory and Communications Universidad Carlos III de Madrid Madrid Spain; 3 Evidence Based Behavior Madrid Spain; 4 Department of Psychiatry University Hospital Jimenez Diaz Foundation Madrid Spain; 5 Gregorio Marañón Health Research Institute Madrid Spain

**Keywords:** anxiety disorder, COVID-19, social media, public health, digital phenotype, ecological momentary assessment, smartphone, machine learning, hidden Markov model

## Abstract

**Background:**

Anxiety symptoms during public health crises are associated with adverse psychiatric outcomes and impaired health decision-making. The interaction between real-time social media use patterns and clinical anxiety during infectious disease outbreaks is underexplored.

**Objective:**

We aimed to evaluate the usage pattern of 2 types of social media apps (communication and social networking) among patients in outpatient psychiatric treatment during the COVID-19 surge and lockdown in Madrid, Spain and their short-term anxiety symptoms (7-item General Anxiety Disorder scale) at clinical follow-up.

**Methods:**

The individual-level shifts in median social media usage behavior from February 1 through May 3, 2020 were summarized using repeated measures analysis of variance that accounted for the fixed effects of the lockdown (prelockdown versus postlockdown), group (clinical anxiety group versus nonclinical anxiety group), the interaction of lockdown and group, and random effects of users. A machine learning–based approach that combined a hidden Markov model and logistic regression was applied to predict clinical anxiety (n=44) and nonclinical anxiety (n=51), based on longitudinal time-series data that comprised communication and social networking app usage (in seconds) as well as anxiety-associated clinical survey variables, including the presence of an essential worker in the household, worries about life instability, changes in social interaction frequency during the lockdown, cohabitation status, and health status.

**Results:**

Individual-level analysis of daily social media usage showed that the increase in communication app usage from prelockdown to lockdown period was significantly smaller in the clinical anxiety group than that in the nonclinical anxiety group (F_1,72_=3.84, *P*=.05). The machine learning model achieved a mean accuracy of 62.30% (SD 16%) and area under the receiver operating curve 0.70 (SD 0.19) in 10-fold cross-validation in identifying the clinical anxiety group.

**Conclusions:**

Patients who reported severe anxiety symptoms were less active in communication apps after the mandated lockdown and more engaged in social networking apps in the overall period, which suggested that there was a different pattern of digital social behavior for adapting to the crisis. Predictive modeling using digital biomarkers—passive-sensing of shifts in category-based social media app usage during the lockdown—can identify individuals at risk for psychiatric sequelae.

## Introduction

During the early peaks of casualties from the first wave of the COVID-19 pandemic, government lockdown measures in urban centers drastically diminished in-person communication and forced individuals to turn to the digital world to connect with others [1]. Physical isolation has been linked with suicidal ideation, depression, and posttraumatic stress disorder during infectious disease outbreaks [2-5]; it can increase the intensity and perception of threat, especially with the inherent uncertainty of a high-mortality novel virus outbreak [6,7]. Anxiety further causes maladaptive coping behavior, such as substance use, which can, in turn, lead to adverse mental health outcomes in a negative feedback loop [8]. Anxiety can also compromise effective social decision-making, which was evident in panic buying, hoarding, and excessive internet searching for information during the COVID-19 pandemic [9,10].

In contrast, positive public health outcomes are driven by individuals’ sound health decisions made based on accurate perceptions of the costs and benefits to self and society [11]. Therefore, remotely identifying the severity of short-term anxiety symptoms in the population during lockdown measures is an important public health agenda. It may lead to early detection of those who are at risk for impaired decision-making, maladaptive coping, and psychiatric sequelae [8].

In recent years, passive smartphone sensor data have been utilized in empirical studies to identify various psychiatric presentations and mental health-related behaviors, including social anxiety severity [12,13]. Current literature on social media and its impact on mental health outcomes provides conflicting perspectives about the role of social media use in the development of anxiety during crises [14,15]. For example, excessive time spent searching for news on social media has been linked with higher anxiety during COVID-19 and Ebola outbreaks [16-18]. In contrast, ready exposure to public health information through social media during the Middle East respiratory syndrome–related coronavirus outbreak was positively related to the formation of appropriate risk perceptions in the population [19]. A previous report [20] from our group suggested that increased usage of social media predicts increased physical activity, possibly promoting healthy behavior during COVID-19–related lockdown. In other words, identifying fine-grained frameworks to describe user behavior on social media platforms, as opposed to simply verifying social media usage, appears to be relevant to gathering important public health information in real time.

In this study, we focused on analyzing daily time spent on apps in 2 social media categories (communication and social networking) in a sample of psychiatric outpatients in Madrid, Spain, before and during the mandatory COVID-19 lockdown. Communication apps allow direct messaging activity, and social networking apps enable interactions on social networking sites in heterogeneous forms. We hypothesized that differential forms of social media app activity can represent the distinct user behaviors that interplay with the manifestation of anxiety. Specifically, we aimed to employ a machine learning model and individual app usage patterns during this period to predict who would report clinical anxiety symptoms at follow-up. 

## Methods

### Study Participants

Data were drawn from 2 ongoing studies [21,22] of psychiatric outpatients (n=142) in Madrid, Spain, that involve remote smartphone monitoring. Both studies received approval from the Institutional Review Board at the Psychiatry Department of *Fundación Jimenez Diaz* Hospital, and all participants provided written informed consent. Participants were required to be aged 18 years or older, fluent in Spanish, and possess a smartphone with internet access.

### Data

Sociodemographic and clinical information were collected from all participants at baseline before the onset of the pandemic via an electronic health tool (MEmind [21,23]). Sociodemographic data included age, gender, household composition, marital status, and employment status. Clinical data entailed International Classification of Diseases, tenth revision, psychiatric diagnoses grouped into the following categories: (1) anxiety, stress, and trauma-related disorders; (2) unipolar or bipolar mood disorders; (3) personality disorders; (4) substance use disorders; (5) psychotic disorders, and (6) other disorders.

From February 1 through May 3, 2020, passive smartphone usage data were collected using eB2 Mindcare [24-26], a clinically validated eHealth platform. On March 14, a country-wide state of emergency was declared due to rising mortality rates from the coronavirus pandemic, and the government mandated a lockdown of all individuals who were not essential workers (ie, they were restricted to their residences, except when purchasing food and medicines or attending emergencies). On May 4, Madrid entered the first step in a de-escalation of the lockdown, which allowed the reopening of small businesses and walking outside within set time slots [27]. Daily time (in seconds) automatically logged on communication apps and social networking apps were extracted and analyzed in the prelockdown (ie, February 1 through March 13) and the lockdown periods (ie, March 14 through May 3). Social media app categories—communication and social networking—were based on the labels designated in the Google App store. Communication apps included messaging, chat/IM, dialer, and browser apps such as WhatsApp, Telegram, Facebook Messenger, and Gmail; social networking apps were primarily those for sites such as Instagram, Twitter, and TikTok [28].

A clinical psychologist collected short-term mental health outcomes, including self-reported intensity of psychosocial stressors during the lockdown and Generalized Anxiety Disorder 7-item scale (GAD-7), by phone follow-up between May 12 and June 3 after the initial lockdown measures had been lifted. Clinical anxiety was defined as a GAD-7 score of 10 or greater, given its diagnostic value in screening for severe GAD, panic disorder, and social phobia [29,30]. COVID-19 exposures, risk perception, and social behaviors during the lockdown period were also assessed during the phone call.

### Statistical Analysis

Group-level differences were evaluated using the 2-sided *z* score, for sample proportions (ICD-10 diagnosis, gender, cohabitation status, coronavirus exposure risk items); the chi-square test, for categorical clinical variables (family, employment, physical health status, worries about life instability, modes of contact and social interaction frequency during the lockdown); or the 2-sided *t* test, for continuous variables (age, GAD-7), with a type I error of 5%. After logarithmically transforming usage data (seconds) spent on communication and social networking apps to normal distributions, individual-level differences in median social media usage during the prelockdown and lockdown periods were summarized using repeated measures analysis of variance that accounted for the fixed effects of the lockdown (prelockdown versus postlockdown), group (clinical anxiety group versus nonclinical anxiety group), the interaction of lockdown and group, and random effects of users. The median was chosen as the estimate of central tendency (instead of the mean) because the distribution of app usage was such that the mean was sensitive to extremely low values and significantly correlated with the number of logged days, which varied among users. To ensure stability when using the point estimate representing the time series variables in prelockdown vs. lockdown, analyses were restricted to the patients whose total logged days in both communication and social networking apps was more than half the total days in the lockdown (≥26 out of 51 days) and prelockdown period (≥22 out of 42 days), and whose median usage was within 2.5 SD of all available users’ data in each period. This filtering resulted in 74 communication app users and 42 social networking app users available for analysis. Missing users were separately analyzed. To investigate the dose-related association of social media app usage with the intensity of anxiety symptoms later reported, Pearson correlations, of GAD-7 scores with median app usage time in the prelockdown and lockdown period, were calculated; we controlled for age given its significant correlation with social media app usage. Differences in the dependent correlations between prelockdown and lockdown periods were analyzed using the Steiger *Z* test [31].

### Machine Learning Models

**Figure 1:**
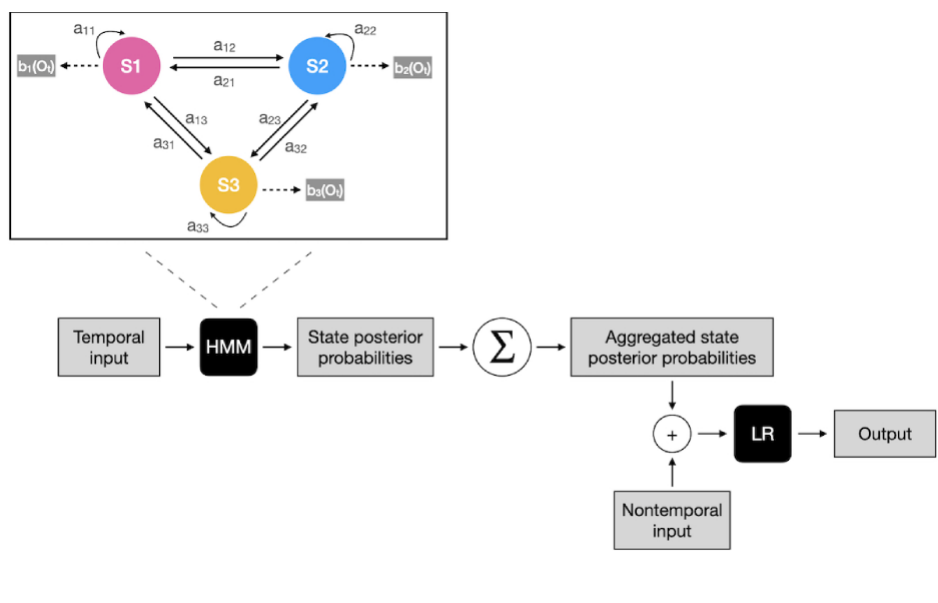
The proposed anxiety prediction pipeline. LR: logistic regression; HMM; hidden Markov model; S1, S2, S3; the 3 states of the hidden Markov model.

We designed a 2-step approach that combined a probabilistic generative model, namely a hidden Markov model (HMM) [32] for temporal data processing and aggregation, with logistic regression to predict the binary outcome (clinical anxiety versus nonclinical anxiety) by dichotomized GAD-7. Nonclinical anxiety outcome (n=51) was encoded as the negative label and clinical anxiety outcome (n=44) was encoded as the positive label. The class imbalance problem was insignificant. Continuous longitudinal daily communication and social networking app usage in seconds were chosen as independent variables, with anxiety-associated clinical variables as additional predictors (Figure 1).

HMMs are commonly used for time-series analysis. HMMs model generative sequences, which are characterized by a set of observable sequences. A first-order Markov chain process generates the states of the HMM. The following components specify an HMM: *S*=*s*_1_*s*_2_...*s_N_*, a set of *N* states; *A*=*a*_11_...*a_NN_*, a transition probability matrix; *O*=*o*_1_*o*_2_...*o_T_*, a sequence of *T* observations; *B*=*b*_1_(*o_t_*), emission probabilities, expressing the probability of an observation *o_t_* being generated from state *i*; and π=π_1_... π*_N_*, an initial probability distribution over states.

The state space of the applied HMM is discrete, while the observations can be discrete or continuous. In this study, communication and social networking app usage are treated as continuous variables from a Gaussian distribution. The parameters of an HMM can be trained with the Baum-Welch algorithm, a variation of the expectation-maximization algorithm. The model can deal with missing data using marginalization without requiring imputation before training. To select the optimal number of hidden states, we computed the Akaike information criterion and the Bayesian information criterion [33] after training HMMs with 2-19 numbers of states.

Once the optimal HMM was selected for each sequence, we computed the state posterior probabilities *P*(*s*_i_ = *k* | *x*) (the probability of being in state *k* at position *i* of the sequence *x*) for each time point and aggregated them by summing over time for each patient. This feature vector of length *N* was then concatenated with nontemporal clinical features of length *N*_clinical_ to form the feature vector of length *N* + *N*_clinical_. Hence the data set of size for the logistic regression was *N*_patients_ × (*N* + *N*_clinical_). Age, gender, self-reported worries about life instability during the lockdown, health status, presence of an essential worker in the household, changes in the frequency of social interactions during quarantine, and current employment status were chosen as nontemporal features for the model training. These features were selected because of the differences between the clinical anxiety and nonclinical anxiety groups and correlations with GAD-7 (Figure S3 in Multimedia Appendix 1). Given the clinical association between social isolation and anxiety disorder in the literature [34] and the impact of the lockdown differently impacting the social media app usage for those living alone in our sample (Table S1 in Multimedia Appendix 1), we additionally included cohabitation status.

The evaluation was performed using *k*-fold cross-validation [34,35], due to the limited data sample. 10 train-test splits were created from the dataset. Similarly, 10 logistic regression models were created and trained for evaluation. Since we had 95 patients, this means that in the first 5 splits, we trained the model on data from 85 patients and tested the model on data from 10 patients, while in the last 5 splits trained the model on 86 patients and tested the model on 9 patients. The results are summarized with a mean and standard deviation of the model accuracy, and area under the receiver operating curve (AUROC) scores.

Finally, we performed feature importance analysis by computing Shapley additive explanations (SHAP) values [36], which provide an overview of important features in the machine learning models by designating the weight of predictability of each feature positively or negatively to the target variable. We averaged the SHAP values over the 10-fold cross-validation for every feature for each patient.

## Results

### Study Participants

Of 142 participants (Table 1) 99 (70%) were female, and the mean age was 45 years (SD 14). The most commonly represented psychiatric diagnostic category was anxiety, trauma, or stress-related disorder, followed by unipolar or bipolar mood disorder. At the postlockdown phone follow-up, the mean anxiety symptom score (GAD-7) was 9.6 (range 0-21, SD 5.5). Of the 142 patients, 1 (1%) tested positive for SARS-CoV-2 (with a polymerase chain reaction test), 16 (11%) were living with people with COVID-19 infections, 6 (4%) were living with the older adults, 43 (30%) lived with essential workers, and 43 (30%) personally knew individuals who died of COVID-19. On a group level, the clinical anxiety group had an 18% higher likelihood of having an essential worker in the household (*z*=2.3, *P*=.02; 95% CI 0.03-0.34) and reported higher intensity of worries about life instability (χ_4_^2^=12, *P*=.01), more negative self-ratings of health status (χ_2_^2^=6.4, *P*=.04), and lower frequency of social interactions (χ_2_^2^=6.8, *P*=.03) than those reported by the nonclinical anxiety group.

**Table 1:** Demographic and clinical information.

Variable				All (n=142)		Clinical anxiety^a^ (n=66)		Nonclinical anxiety^b^ (n=76)		*z* score, *t* test statistic (*df*)^c^, or chi-square (*df*)^d^			*P* value	
**Baseline sociodemographic and clinical information**														
	Age (years), mean (SD)		45 (14.2)		43 (13.6)		47 (14.6)		–1.8 (139)^c^			.07		
	**Gender, n (%)**										0.36			.72
		Male	43 (30)		19 (29)		24 (32)							
		Female	99 (70)		47 (71)		52 (68)							
	**Cohabitating, n (%)**								0.83			.40		
		No	21 (15)		8 (12)		13 (17)							
		Yes	121 (85)		58 (88)		63 (83)							
	**Family status, n (%)**										3.5 (3)^d^			.32
		Single	46 (32)		21 (32)		25 (33)							
		Separated	26 (18)		11 (17)		15 (20)							
		Widowed	6 (4)		5 (8)		1 (1)							
		Married or cohabitating for >6 months	64 (45)		29 (44)		35 (46)							
	**Employment status, n (%)**										7.5 (5)^d^			.18
		Employed, student or homemaker	51 (36)		23 (35)		28 (37)							
		Unemployed without subsidy	28 (20)		9 (14)		19 (25)							
		Unemployed with subsidy	17 (12)		8 (12)		9 (12)							
		Long-term disability	11 (8)		7 (11)		4 (5)							
		Temporarily incapacitated	26 (18)		16 (25)		10 (13)							
		Retired	8 (6)		2 (3)		6 (8)							
	Anxiety, stress, or trauma disorder^e^, n (%)		79 (58)		41 (63)		38 (54)		1.1			.26		
	Mood disorder^e^, n (%)		50 (37)		28 (43)		22 (31)		1.5			.14		
	Personality disorder^e^, n (%)		30 (22)		14 (22)		16 (23)		–0.14			.89		
	Substance use disorder^e^, n (%)		8 (6)		5 (8)		3 (4)		0.86			.39		
	Psychotic disorder^e^, n (%)		3 (2)		2 (3)		1 (1)		0.66			.51		
	Other psychiatric disorder^e^, n (%)		21 (15)		10 (15)		11 (15)		–0.02			.99		
**Risk perception and social behaviors during the pandemic lockdown**														
	**Worries about life instability during lockdown, n (%)**										12 (4)^d^			.01
		Not at all	21 (15)		8 (12)		13 (18)							
		Slightly	32 (23)		9 (14)		23 (31)							
		Moderately	37 (26)		19 (29)		18 (24)							
		A lot	35 (25)		18 (27)		17 (23)							
		Extremely	15 (11)		12 (18)		3 (4)							
	**Self-ratings of physical health, n (%)**										6.4 (2)^d^			.04
		Positive	66 (46)		23 (35)		43 (57)							
		Regular	56 (40)		32 (49)		24 (32)							
		Negative	19 (13)		10 (15)		9 (12)							
	**Modes of contact with outside, n (%)**										3.2 (2)^d^			.21
		Phone calls	66 (46)		34 (52)		32 (43)							
		Video calls	45 (32)		16 (24)		29 (39)							
		Messengers (WhatsApp, Telegram, etc)	29 (20)		15 (23)		14 (19)							
	**Changes in frequency of social interactions during lockdown, n (%)**										6.8 (2)^d^			.03
		Less frequent than prepandemic	63 (44)		36 (55)		63 (36)							
		More or less the same	48 (34)		21 (32)		48 (36)							
		More frequent than prepandemic	31 (22)		9 (14)		31 (29)							
	Tested positive on SARS-CoV-2 PCR^f^ test, n (%)		1(1)		0 (0)		1 (1)		–0.94			.34		
	Living with people with COVID-19, n (%)		16 (11)		9 (14)		7 (10)		0.75			.45		
	Living with older adult, n (%)		6 (4)		3 (5)		3 (4)		0.20			.84		
	Essential workers in household, n (%)		43 (30)		27 (41)		16 (23)		2.3			.02		
	Knew people who died of COVID-19, n (%)		43 (30)		25 (38)		18 (24)		1.7			.08		
	Generalized Anxiety Disorder-7 score postlockdown (mean, SD)		9.6 (5.5)		14.6 (3.1)		5.2 (2.8)		19 (133)^c^			<.001		

^a^Generalized Anxiety Disorder-7 score ≥10.

^b^Generalized Anxiety Disorder-7 score <10.

^c^A 2-sided *t* test was used.

^d^A chi-square independence test was used.

^e^Psychiatric diagnosis categories are not mutually exclusive.

^f^PCR: polymerase chain reaction.

Active users (n=42; Table S1 in Multimedia Appendix 1) of social networking apps whose usage was consistent during the analysis period had 21% higher likelihood of carrying anxiety, stress, or trauma-related disorder diagnoses (*z*=2.2, *P*=.03; 95% CI 0.03-0.37); were approximately 8 years younger (t_73_=–3.2, *P*=.002; 95% CI 3.0-14); had 14% higher likelihood of cohabitation (*z*=–2.2, *P*=.03; 95% CI 0.04-0.24); and had a higher likelihood of being employed, students, or homemakers (χ_2_^2^=13, *P*=.02), than missing or inconsistent users (n=100). However, there was no significant difference in GAD-7 (t_88_=0.75, *P*=.46), which was our primary outcome for labeling the clinical anxiety group in the prediction model. Missing or inconsistent users (n=68; Table S1 in Multimedia Appendix 1) in the communication apps were not significantly different from the active users (n=74) in anxiety, stress, or trauma-related disorder diagnoses (*P*=.50), age (*P*=.41), cohabitation status (*P*=.36), employment status (*P*=.29), or GAD-7 (*P*=.34).

### Statistical Analysis of Social Media Use Across Anxiety Groups and Periods

**Figure 2:**
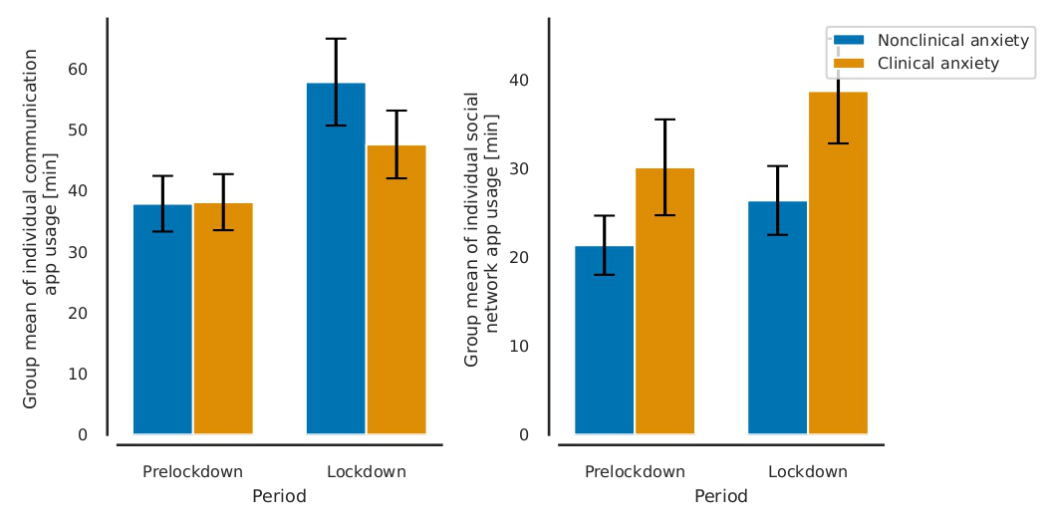
The effect of lockdown on the increase in communication app usage was lower in the clinical anxiety group. Error bars indicate 95% standard error of the mean.

From prelockdown to lockdown period, the mean of individual median usage on communication app (in both anxiety groups) increased from 29 minutes (95% CI 25-35) to 41 minutes (95% CI 35-49; *F*_1,72_=26; *P*<.001), and usage on social networking app increased from 19 minutes (95% CI 14-25) to 25 minutes (95% CI 20-32; *F*_1,40_=13; *P*<.001) (Figure 2; Table S2 in Multimedia Appendix 1). There was a significant interaction of group and lockdown period, such that communication app usage was not significantly different between groups prelockdown but increased significantly more in the nonclinical anxiety group during the lockdown (from 29 minutes to 46 minutes, 95% CI 37-57) than that in the clinical anxiety group (from 30 minutes to 37 minutes; 95% CI 29-46; *F*_1,72_=3.8; *P*=.05). There was no main effect of group on communication app usage in the entire period. There was a trend in the main effect of group on social networking app in the entire period such that the clinical anxiety group had higher median usage at 27 minutes (95% CI 22-33) than that of the nonclinical anxiety group at 17 minutes (95% CI 13-23), but it was not significant (*F*_1,40_=3.4; *P*=.07).

No significant correlations were found between GAD-7 and median communication or social networking app usage during prelockdown (communication: *P*=.59; social networking: *P*=.24), lockdown (communication: *P*=.14; social networking: *P*=.11), and the entire period (communication: *P*=.27; social networking: *P*=.14) (Figure S4 and Table S3 in Multimedia Appendix 1). There was no statistically significant impact of the period on dependent correlations in the patients (Table S3 in Multimedia Appendix 1, Steiger *Z* test; *P*=.25 in communication app; *P*=.47 in social networking app).

### Machine Learning Pipeline for Predicting Clinical Anxiety Group

Only the patients with communication and social networking app usage data during both the prelockdown (≥1 out of 42 days) and lockdown period (≥1 out of 51 days) were considered for the model training. This resulted in 95 patients in the model with varying sequences of individual app usage data. In these sequences, 8.76% (655/7476) of the communication app and 30.26% (2262/7476) of the social networking app usage data were missing in the data set. Figure S4 in Multimedia Appendix 1 shows the overall data distribution grouped by the anxiety type.

**Figure 3:**
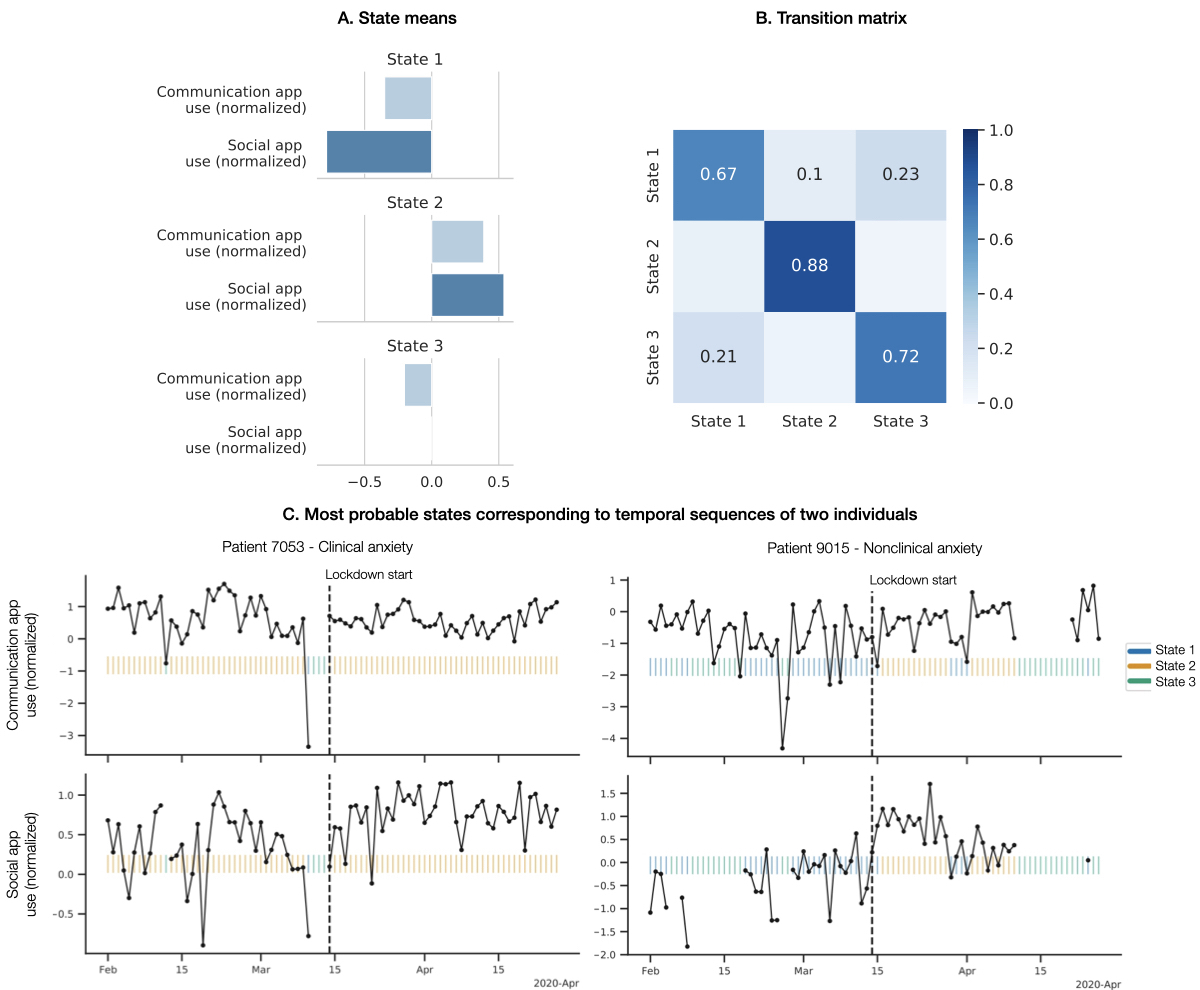
The 3-state hidden Markov model parameters used for temporal data modeling and most probable hidden Markov model states applied to daily communication and social media app usage of example individuals with clinical and nonclinical anxiety. Temporal variables were normalized before model training, providing the negative means. Large state transition probabilities suggest that the states were relatively stable.

An HMM with 3 hidden states (Figure 3A and Figure 3B) proved to capture the underlying data patterns the best according to the Akaike information criterion and Bayesian information criterion analysis and also led to the most interpretable states. State 2 was the most stable (self-transition probability of 0.88), while transitioning between states 1 and 3 was more likely. State 3 captured days with relatively low communication app usage and average social networking usage in the sample, while states 1 and 2 captured days with lower and higher app usage, respectively. When applied to individual observation sequences, state 3 preferentially represented the missing observations (ie, days the apps were not consistently used). State 2 preferentially represented the days of active and consistent social media usage, and state 1 preferentially represented the days of still active (but less so) and volatile usage (Figure 3C). For example, for patient 7053 with clinical anxiety, most days were in state 2, punctuated with 3 missing/inactive days (state 3), and after the lockdown social networking app usage increased. In the case of patient 9105 with nonclinical anxiety, days after the lockdown were marked with increased communication app usage (state 2), but during the overall period social networking app usage was less, capturing missing (state 3) and inactive or volatile (state 1) days.

Our model achieved a mean accuracy of 62.30% (SD 16%) and an AUROC of 0.70 (SD 0.19) in predicting the clinical anxiety group on the test sets (Table S4 in Multimedia Appendix 1). Performance metrics show that the model performs well on the majority of the splits; however, it underperforms on splits 6, 7, and 10, due in part to the nonrepresentative demographic features for the clinical anxiety group (Figure S5 in Multimedia Appendix 1). For example, in split 10, which had the lowest predictability with an AUROC of 0.40, clinical anxiety individuals had atypical risk perception (only 1 individual reported the presence of essential workers in the household) or self-report patterns (reported clinical anxiety despite having relatively good health and few worries about life instability during the lockdown) (Figure S6 in Multimedia Appendix 1).

**Figure 4:**
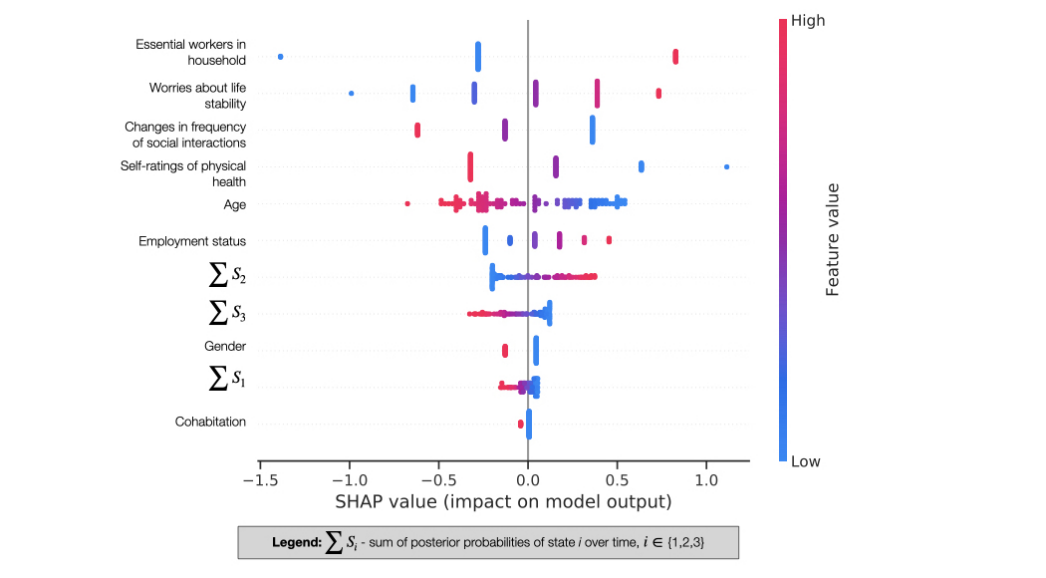
Feature importance (Shapley additive explanation [SHAP] value) for the logistic regression model trained for the anxiety prediction task in the order of descending importance (colored by value, from low to high). Each point is for a feature and an instance, and overlapping points are jittered in the y-axis direction. SHAP values encode the feature’s predictability for classifying the participant (positive, in the clinical anxiety group; negative, in the nonclinical anxiety group). For explanation of the encoded feature values see Table S5 in Multimedia Appendix 1.

The majority of nontemporal features, led by the presence of essential workers in the household, outweighed the aggregated representation of the temporal features in importance. Among temporal features, the aggregated posterior probability of state 2 (higher social networking app use) was the most important predictor of the clinical anxiety group (Figure 4), consistent with our missing user analysis, where active social networking app users had a higher burden of anxiety-related diagnoses (Table S1 in Multimedia Appendix 1). Despite their lower feature importance, states 1 and 3 still provided important insight into users’ longitudinal behavior such that inactive and/or volatile social media usage patterns, specifically in lower communication app usage, predicted the clinical anxiety group. This is also consistent with our finding that clinical anxiety group communication app use was significantly lower during the lockdown period (*P*=.05; Figure 2).

## Discussion

### Principal Findings

Our findings demonstrate that among active social media users, those who reported clinical levels of anxiety symptoms after the mandatory lockdown spent less time on communication apps during the lockdown. Active social-networking app users, biased toward younger patients, additionally had a higher likelihood of having an anxiety disorder diagnosis. Our machine learning–based model, trained on the temporal series of communication and social networking app usage and clinically important features of self-report and demographic variables, accurately predicted which individuals were in the clinical anxiety group from higher social networking app usage and lower communication app usage. Our machine learning–based model results suggest that passive tracking of decreased communication app usage and increased social networking app usage through the lockdown period can predict users reporting clinical anxiety symptoms, at risk for impaired decision-making, maladaptive coping, and psychiatric sequelae during public health crises and lockdown periods [8]. Early remote detection of at-risk individuals would allow limited mental health resources to be allocated to serve those with the highest need and prevent or reduce negative mental health outcomes.

We interpret the findings from the perspective that patients who reported fewer anxiety symptoms proactively harnessed their digital social environment by using communication apps (eg, WhatsApp and Telegram) to initiate contact or respond to others’ direct messages during this highly anxiogenic period (ie, during the COVID-19 pandemic and related lockdown). Our analysis is consistent with the clinical anxiety group’s self-reports that they had less frequent social interactions with others during the lockdown. Social support systems, either in-person or online, are well-known protective factors against physiological and psychological stressors and can mitigate the impact of loneliness in times of uncertainty, including during infectious disease outbreaks [37-41]. Decreased social support has been associated with a higher mental health burden in COVID-19 literature [42,43]. Especially during mandated quarantine, when in-person contact is significantly limited, social media engagement can be viewed as a potentially healthy adaptive mechanism to regulate negative affect [44,45].

Conversely, users in our sample who were highly active on social networking apps were more likely to be diagnosed with anxiety disorder and report clinical anxiety symptoms. Social networking apps, such as Facebook, Twitter, TikTok, and Instagram, are examples of web 2.0 technology apps that have shifted the recent web-based environment of health communications, from traditionally one-way communication to interactive and iterative, characterized by passive sharing, active collaboration, and amplification of information [46,47]. However, public digital space can expose users to unfiltered or anonymous information that promotes fear during a health crisis [48] and has been linked, as a source of COVID-19 conspiracy theories and a sign of complex social and medical needs among patients, with a number of high emergency room visits [49,50]. Our analysis suggests that active engagement on social networking apps is a marker of anxiety that may be associated with individual behavioral traits, perhaps activated by increasing risk perceptions of the virus and psychosocial stressors of the lockdown period.

Our passively sensed user-driven data were prospectively collected within users’ natural environment and under no influence of perceived experimental manipulation. They also contained clearly divided timeframes, followed by timely clinical surveys, which allowed our model of human behavior during the national lockdown to have high interpretability, which is critical for translating digital phenotyping research to real-life application [51]. User-driven passive data collection also reduced sampling bias and web-based activity measurement bias, particularly in self-reported scales [52]. The machine learning model utilized a data-driven algorithm to predict the clinical anxiety group, addressing missing observations and changes in social media usage after the lockdown. Although social networking app users were younger and had a higher burden of anxiety disorder diagnosis, there was no sampling bias of clinical or demographic variables in the communication app users. Our sample overall was a diverse cohort of psychiatric patients with varying ages, diagnoses, health, employment, and marital status. Therefore, our data are highly generalizable to populations of psychiatry outpatients and provide clinical utility by elucidating the link between digital behavior and public mental health outcomes in the real world [53].

### Limitations

Our analysis was based on observing a small number of patients and should be interpreted with the following limitations. First, the data cannot explain the causal link between app usage and the severity of anxiety. For example, we do not know if decreased engagement in communication apps contributed to the reporting of higher anxiety symptoms, or if the former was a characteristic of the group that developed short-term clinical anxiety symptoms during the crisis (ie, a smaller volume of social support for communication to begin with). Second, besides *general worries about life instability during the lockdown*, there were no other independent variables that may reflect the evolution of subjective emotions included in the model to predict the anxiety states at clinical follow-up. Study participants had a daily mood self-reporting option on their smartphones, but such reporting was entirely voluntary, and mood data were largely missing during the lockdown. We acknowledge that our study participants were in an unprecedented and anxiogenic natural circumstance at the time. The lockdown likely increased the anxiety and stress levels of all users (mean GAD-7 was 9.6, with a clinical cut-off of 10), and we had not collected their baseline GAD-7 before the lockdown, in order to make a comparison. Therefore, the utility of our model is limited to detecting those with clinical severity anxiety symptoms (ie, GAD-7≥10). We suggest that future data-based anxiety prediction research should include self-reports of anxiety at multiple time points to improve model accuracy. Third, our assumption of user behavior was limited to the descriptive nature of the app category (communication apps require direct messaging activity and are generally used within a known social circle; social networking apps allow simple browsing of the others’ contents and provides ready exposure to anonymous content). However, the extent of the complex interplay between social media behavior and user intention is unlikely to be captured via passive sensing of total time spent on app categories. For example, although our study participants confirmed at the clinical interview that they used communication apps such as WhatsApp to stay in touch with others during the lockdown, there are reported benefits of actively using social networking sites, such as Instagram and TikTok to keep in touch or even to promote mental health awareness [54]. Further quantitative and qualitative analyses are needed to understand the mental health implications of these 2 app categories. We believe that analyzing multimedia input and output by emotional valence of content, types (text, audio, and video messages), the direction of messaging (is the user initiating or receiving social media activity), and the audience (is the user interacting with one person or multiple anonymous) will be relevant to test our behavioral hypothesis of the anxiety-relieving and anxiety-promoting effects of social media use. The privacy and patient confidentiality terms in our research protocol and data sharing protocol by third app parties prohibited collecting such information in this study.

### Conclusions

To the best of our knowledge, our empirical data are the first to suggest that category-based passive sensing of a shift in smartphone usage patterns can be markers of clinical anxiety symptoms. Further studies, to digitally phenotype short-term reports of anxiety using granular behaviors on social media, are necessary for public health research when in-person psychiatric evaluations are limited during mandated physical isolation.

## Appendix

Multimedia Appendix 1 Supplementary tables and figures for statistical data analysis and the 10-fold cross-validation of our hidden Markov + logistic regression model.

## Abbreviations

AUROC: area under the receiver operating characteristics curve
GAD-7: Generalized Anxiety Disorder scale
HMM: hidden Markov model
SHAP: Shapley additive explanation
