# Supplementary material for: Shift in Social Media App Usage During COVID-19 Lockdown and Clinical Anxiety Symptoms: Machine Learning–Based Ecological Momentary Assessment Study
Source: JMIR Ment Health. 2021 Sep 15;8(9):e30833. doi: 10.2196/30833 (PMC8448085; doi:10.2196/30833)
Supplement: Multimedia Appendix 1 [file mental_v8i9e30833_app1.pdf]

Multimedia Appendix 1

Table S1: Missing user analysis. Active social network app users have a higher burden of anxiety, stress, and trauma-related disorder diagnoses. Data represent N and percentage of available diagnoses unless otherwise specified, between missing or inconsistent users (social network app; n=100, communication app; n=68), i.e. time logged are either fewer than 22 days during the 44 days of the pre-lockdown period, or fewer than 26 days during the 51-days of lockdown period, and active users (social network app; n=42, communication app; n=74, greater than 22 days in pre-lockdown and greater than 26 days in lockdown). Psychiatric diagnosis categories are not mutually exclusive. Statistically significant differences between the clinical anxiety and nonclinical anxiety groups were obtained by two-sided t-test for age, GAD-7; two-sided z-test for gender, cohabitation status, coronavirus exposure risk items, and diagnosis; chi-square independence test for all other categorical variables. Pairwise nominal independence test shows that the significance in chi-square test in employment status variable is driven by an employee, student, or homemaker ~ unemployed without benefits and ~ retired, *P*=.05, .03 respectively).

Social Network App				
Variable	*Missing (n=100)*	*Active (n=42)*	*Statistics (df)*	*P-value*
ICD-10 diagnosis				
Anxiety, stress, or trauma disorder	50 (52%)	29 (73%)	2.2	.03
Mood disorder	36 (38%)	14 (35%)	-0.28	.78
Personality disorder	20 (21%)	10 (25%)	0.53	.59
Substance use disorder	6 (6%)	2 (5%)	-0.28	.78
Psychotic disorder	2 (2%)	1 (3%)	0.15	.88
Other psychiatric disorder				
Eating Disorder	3 (5%)	3 (10%)	0.92	.36
Impulse Control Disorder	1 (2%)	0 (0%)	-0.71	.48
ADHD	2 (3%)	1 (3%)	0.01	.99
Tic disorder	1 (2%)	1 (3%)	0.52	.60
Miscellaneous (e.g. Conversion Disorder)	3 (5%)	5 (16%)	1.9	.06
Generalized Anxiety Disorder-7 Scale (mean, SD)	9.4 (5.7)	10.1 (5.0)	0.75 (88)	.45
Age (mean, SD)	48 (13.5)	39 (14.4)	-3.2 (73)	.002
Gender				
Male	31 (31%)	12 (29%)	0.29	.77
Female	69 (69%)	30 (71%)		
Cohabitation				
No	19 (19%)	2 (5%)	-2.2	.03
Yes	81 (81%)	40 (95%)		
Family Status				
Single	26 (26%)	20 (48%)	6.4 (3)	.09
Separated	20 (20%)	6 (14%)		

Widowed	5 (5%)	1 (2%)		
Married or Cohabitation for >6 months	49 (49%)	15 (36%)		
Employment Status			13 (5)	.02
Employed, student, or homemaker	27 (27%)	24 (57%)		
Unemployed without subsidy	22 (22%)	6 (14%)		
Unemployed with subsidy	14 (14%)	3 (7%)		
Long-term disability	8 (8%)	3 (7%)		
Temporarily incapacitated	20 (20%)	6 (34%)		
Retired	8 (8%)	0 (0%)		
Worries about Life Instability			2.6 (4)	.63
Not at all	14 (14%)	7 (17%)		
Slightly	23 (23%)	9 (21%)		
Moderately	25 (25%)	12 (29%)		
A lot	23 (23%)	12 (29%)		
Self-ratings of Physical Health			3.7 (2)	.16
Positive	45 (45%)	21 (51%)		
Regular	38 (38%)	18 (44%)		
Negative	17 (17%)	2 (5%)		
Modes of Contact with Outside People			0.58 (2)	.75
Phone Calls	45 (45%)	21 (51%)		
Video Calls	32 (32%)	13 (32%)		
Messengers (Whatsapp, etc)	22 (22%)	7 (17%)		
Changes in Frequency of Social Interactions			0.78 (2)	.67
Much to little less	42 (42%)	21 (50%)		
More or less same	35 (35%)	13 (31%)		
Little to much more	22 (22%)	8 (19%)		
Coronavirus (SARS-CoV-2) Exposure Risk				
Tested positive PCR test	1 (1%)	0 (0%)	-0.65	.51
Living with people with coronavirus	9 (9%)	7 (17%)	1.25	.21
Living with elderly	5 (5%)	1 (2%)	-0.70	.48
Essential workers in household	30 (32%)	13 (31%)	-0.07	.94
Knew people who died of COVID-19	30 (30%)	13 (32%)	0.16	.87

	Communication App			
Variable	*Missing (n=68)*	*Active (n=74)*	*Statistics (df)*	P-value
ICD-10 diagnosis				
Anxiety, stress, or trauma disorder	37 (55%)	42 (61%)	0.67	.50
Mood disorder	24 (36%)	26 (38%)	0.22	.82
Personality disorder	15 (22%)	15 (22%)	0.09	.93
Substance use disorder	5 (7%)	3 (4%)	-0.77	.44
Psychotic disorder	1 (1%)	2 (3%)	0.56	.58
Other psychiatric disorder				
Eating Disorder	3 (7%)	3 (6%)	-0.33	.74
Impulse Control Disorder	1 (2%)	0 (0%)	-1.1	.25
ADHD	1 (2%)	2 (4%)	0.37	.71
Tic disorder	0 (0%)	2 (4%)	1.3	.21
Miscellaneous (e.g. Conversion Disorder)	1 (2%)	7 (13%)	1.9	.06
Generalized Anxiety Disorder-7 Scale (mean, SD)	9.1 (5.7)	10.0 (5.0)	0.95 (136)	.34
Age (mean, SD)	46 (13.6)	44 (14.9)	-0.83 (140)	.41
Gender			0.22	.83
Male	20 (29%)	23 (31%)		
Female	48 (71%)	51 (69%)		
Cohabitation			-0.92	.36
No	12 (18%)	9 (12%)		
Yes	56 (82%)	65 (88%)		
Family Status			2.5 (3)	.47
Single	18 (26%)	28 (38%)		
Separated	15 (22%)	11 (15%)		
Widowed	3 (4%)	3 (4%)		
Married or Cohabitation for >6 months	32 (47%)	32 (43%)		
Employment Status			6.1 (5)	.29
Employed, student, or homemaker	21 (31%)	30 (41%)		
Unemployed without subsidy	14 (21%)	14 (19%)		
Unemployed with subsidy	11 (16%)	6 (8%)		
Long-term disability	4 (6%)	7 (9%)		
Temporarily incapacitated	11 (16%)	15 (20%)		
Retired	6 (9%)	2 (3%)		
Worries about Life Instability			5.9 (4)	.21
Not at all	8 (12%)	13 (18%)		
Slightly	17 (25%)	15 (21%)		
Moderately	15 (22%)	22 (30%)		
A lot	16 (24%)	19 (26%)		
Self-ratings of Physical Health			1.3 (2)	.53

Positive	29 (43%)	37 (51%)		
Regular	28 (41%)	28 (38%)		
Negative	11 (16%)	8 (11%)		
Modes of Contact with Outside People			1.5 (2)	.46
Phone Calls	31 (46%)	35 (48%)		
Video Calls	25 (37%)	20 (28%)		
Messengers (Whatsapp, etc)	12 (18%)	17 (24%)		
Changes in Frequency of Social Interactions			1.3 (2)	.53
Much to little less	29 (43%)	34 (46%)		
More or less same	26 (38%)	22 (30%)		
Little to much more	13 (19%)	18 (24%)		
Coronavirus (SARS-CoV-2) Exposure Risk				
Tested positive PCR test	1 (1%)	0 (0%)	-1.1	.29
Living with people with coronavirus	8 (12%)	8 (11%)	-0.21	.83
Living with elderly	3 (5%)	3 (4%)	-0.13	.90
Essential workers in household	21 (32%)	22 (31%)	-0.22	.83
Knew people who died of COVID-19	20 (30%)	23 (32%)	0.21	.83

Figure S1. (a, b) The distribution of communication app usage transformed logarithmically in the sample in both periods (n=142) was long-tailed on the left, making the mean sensitive to extreme values. (c) The number of days apps were logged was correlated with the mean estimate of the usage per individual (Pearson's correlation: 0.16, df=140, *P*=.05) and (d) long-tailed to the left during the entire analyzed period (median 68 days, indicated by the black vertical line).

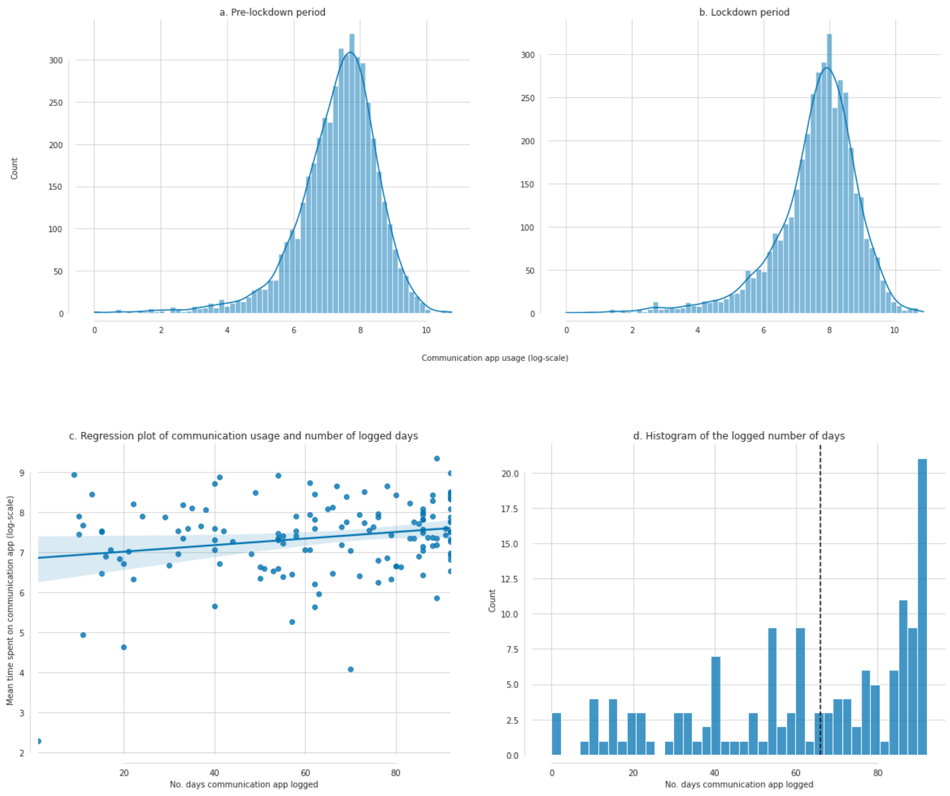

Figure S2. Pearson's correlations between social media app usage across all periods and selected clinical features, i.e. GAD-7 and age in the patient population selected for smartphone data ML analysis (n=95). There was a highly significant negative correlation between social network app usage and age (r=-0.46, *P*<.0001). There was a negative correlation between the communication app usage and GAD-7 (r=-0.16, *P*=.12), as well as age and GAD-7 (r=-0.16, *P*=.13). There was a positive correlation between social network app usage and GAD-7 (r=0.12, *P*=.23).

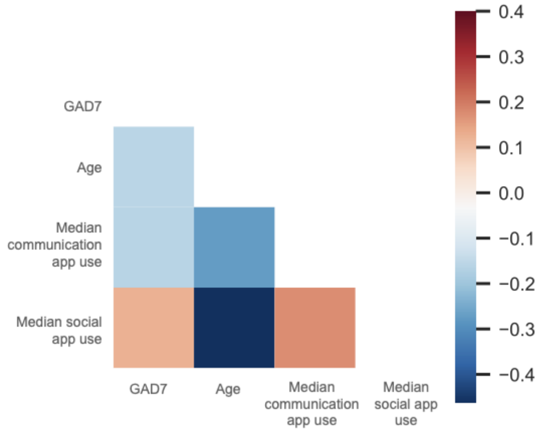

Figure S3. (a-c) Box plots for the variance of GAD-7 scores across the self-reported responses of participants (n=142) regarding their employment status, physical health, and worries about life instability. One-way analyses of means, which did not assume equal variances, followed by pairwise comparisons with non-pooled standard deviation showed significant between-group variances in current activity, $F_{5,37}$ = 2.7, *P*=.03; Retired ~ Temporary disability, *P*=.056; in self-ratings of health status, $F_{3,27}$ =3.2, *P*=.04; Bad ~ Good, *P*=.04; and in worries about life instability, $F_{4,55}$ =5.0, *P* =.002; Extremely ~ Not at all, slightly, moderately (*P* = .03, .003, .03, respectively), A lot ~ slightly, *P* =.03.

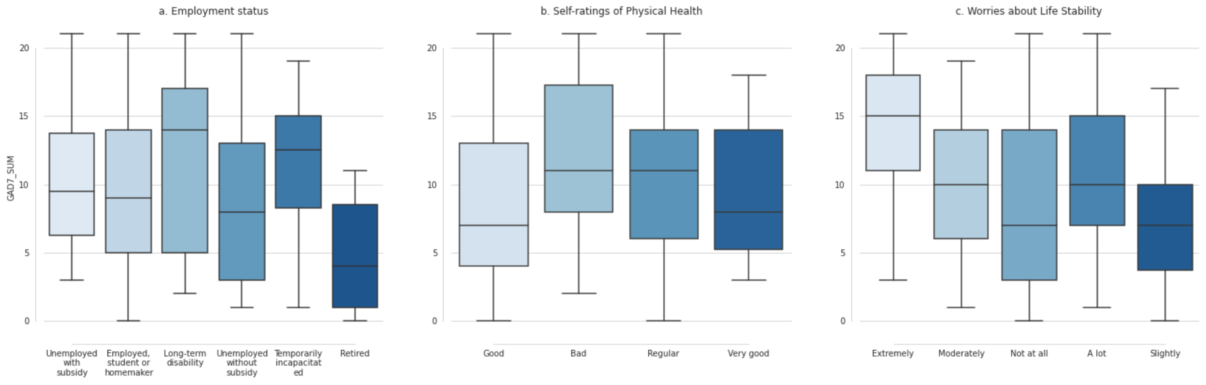

Table S2. Results of repeated ANOVA on logarithmically transformed app usage data to assess the impact of clinical anxiety-group (GAD- 7>=10) and period (time-series data after March 14 only) on individual median app usage in communication and social network categories.

Effects	*F-statistic (df)*	*P-value*	*App Category*
Group	0.47 (72)	.50	Communication
Period	26 (72)	<.001	
Group x Period	3.8 (72)	.05	
Group	3.4 (40)	.07	Social Network
Period	13 (40)	<.001	
Group x Period	0.01 (40)	.92	

Figure S4. (a-b) There was no significant association between GAD-7 scores and median time spent on the app (*P*>.05) in the consistent communication app users (n=74), or social network app users (n=42) (Table S3). Pearson's partial correlations were corrected with age. Steiger's Z test comparing the differences between correlations in distinct periods indicated that there was no significant impact of time on the users' associations.

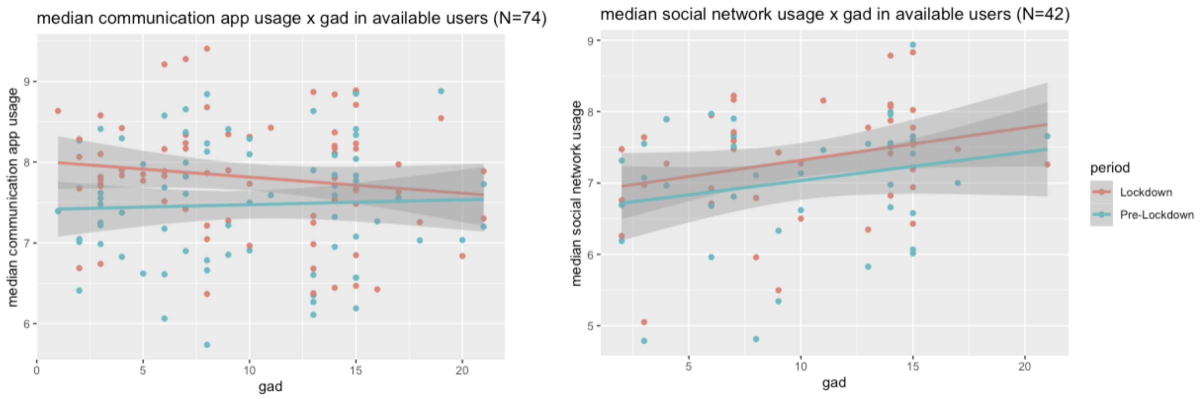

Table S3. Sub-analysis of correlation between app usage and GAD-7 by period, and Steiger's Z test results to test the differences in the dependent correlations.

App	*Period*	*n*	*Pearson's partial correlation*	*P-value*	*Test of difference between two correlations (Steiger's Z)*
Communication	Whole	74	-0.13	.27	
	Pre-lockdown	74	-0.06	.59	t=1.16, *P*=.25 (two-tailed)
	Lockdown	74	-0.17	.14	
Social Network	Whole	42	0.23	.14	t=-0.73, *P*=.47 (two-tailed)
	Pre-lockdown	42	0.19	.24	
	Lockdown	42	0.26	.11	

Figure S5: Distribution of temporal (communication and social network app usage from Feb 1, 2020, through May 4, 2020) (mean and 95% confidence interval) and static variables in the data set consisting of the patients (n=95) are grouped by anxiety. A pronounced increase in time logged on communication apps in the nonclinical anxiety group (GAD-7<10) versus clinical anxiety group (GAD-7>10) after March 14, and increased overall time logged on social network apps in the clinical anxiety group. Usage data collected in seconds were logarithmically transformed and scaled.

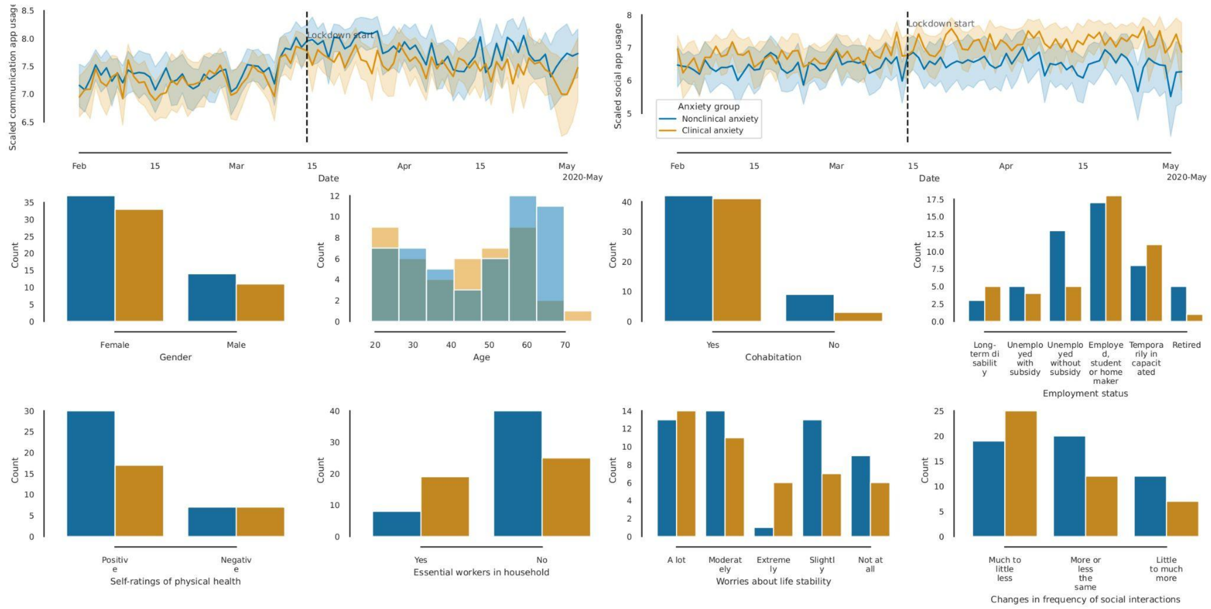

Table S4. Achieved accuracy and area under the receiver operating curve (AUC-ROC) in the 10-fold cross-validation of our HMM+LR model.

**Fold**	**Accuracy (%)**		**AUC-ROC**	
	**Training set**	**Test set**	**Training set**	**Test set**
1	53.61	87.50	0.67	0.88
2	70.57	60.00	0.80	0.76
3	69.40	60.00	0.79	0.76
4	69.51	80.00	0.77	0.88
5	69.20	70.00	0.77	0.84
6	74.23	45.00	0.81	0.50
7	76.90	47.50	0.80	0.65
8	66.96	77.50	0.77	0.80
9	74.73	55.00	0.80	0.60
10	75.82	40.00	0.81	0.30
**Mean (SD)**	70.10 (6.70)	62.30 (16.00)	0.78 (0.04)	0.70 (0.19)

Figure S6. Data distribution in the different splits of the 10-fold cross-validation of our HMM+LR model.

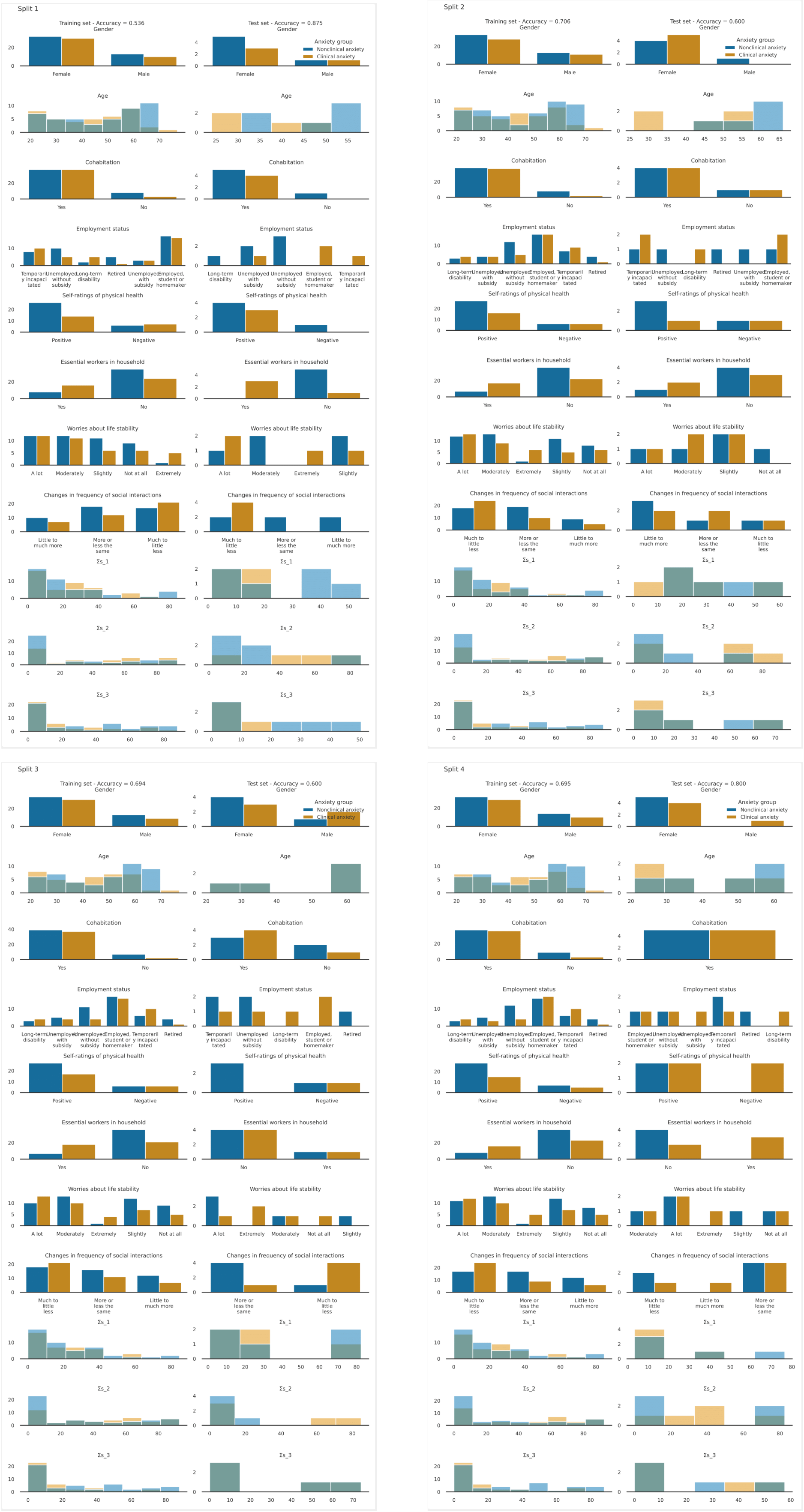

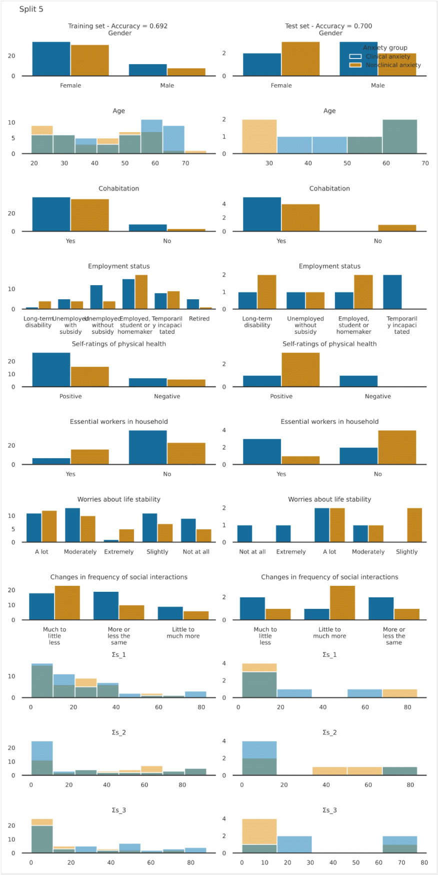

Split 5
Training set - Accuracy = 0.692
Test set - Accuracy = 0.700
Gender
Anxiety group
Clinical anxiety
Nonclinical anxiety
Age
Cohabitation
Employment status
Self-ratings of physical health
Essential workers in household
Worries about life stability
Changes in frequency of social interactions
Σs_1
Σs_2
Σs_3

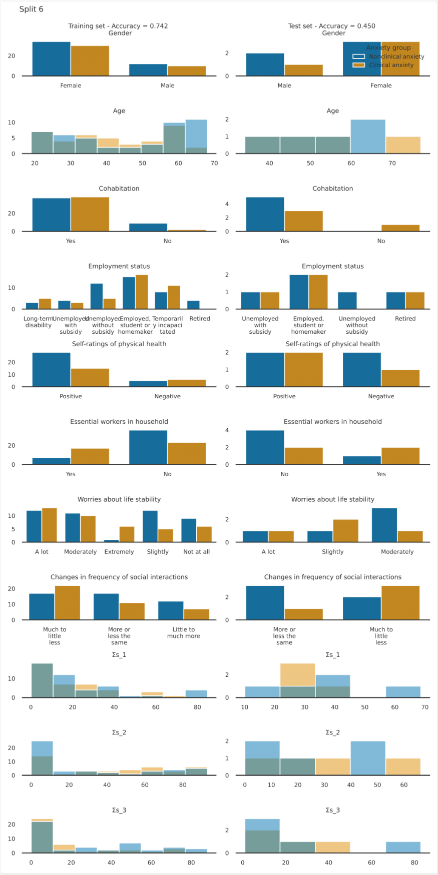

Split 6
Training set - Accuracy = 0.742
Test set - Accuracy = 0.450
Gender
Anxiety group
Nonclinical anxiety
Clinical anxiety
Age
Cohabitation
Employment status
Self-ratings of physical health
Essential workers in household
Worries about life stability
Changes in frequency of social interactions
Σs_1
Σs_2
Σs_3

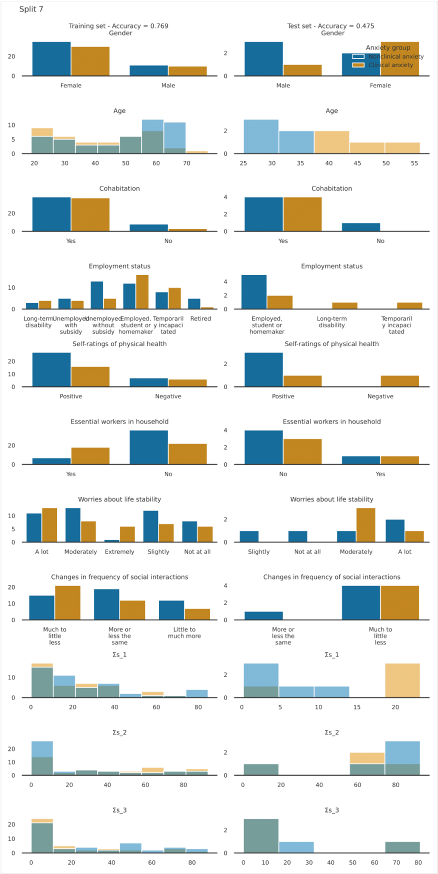

Split 7
Training set - Accuracy = 0.769
Test set - Accuracy = 0.475
Gender
Anxiety group
Nonclinical anxiety
Clinical anxiety
Age
Cohabitation
Employment status
Self-ratings of physical health
Essential workers in household
Worries about life stability
Changes in frequency of social interactions
Σs_1
Σs_2
Σs_3

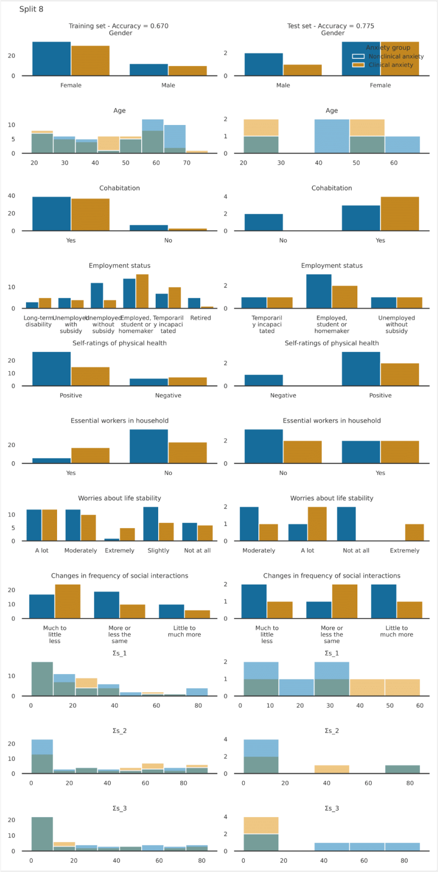

Split 8
Training set - Accuracy = 0.670
Test set - Accuracy = 0.775
Gender
Anxiety group
Nonclinical anxiety
Clinical anxiety
Age
Cohabitation
Employment status
Self-ratings of physical health
Essential workers in household
Worries about life stability
Changes in frequency of social interactions
Σs_1
Σs_2
Σs_3

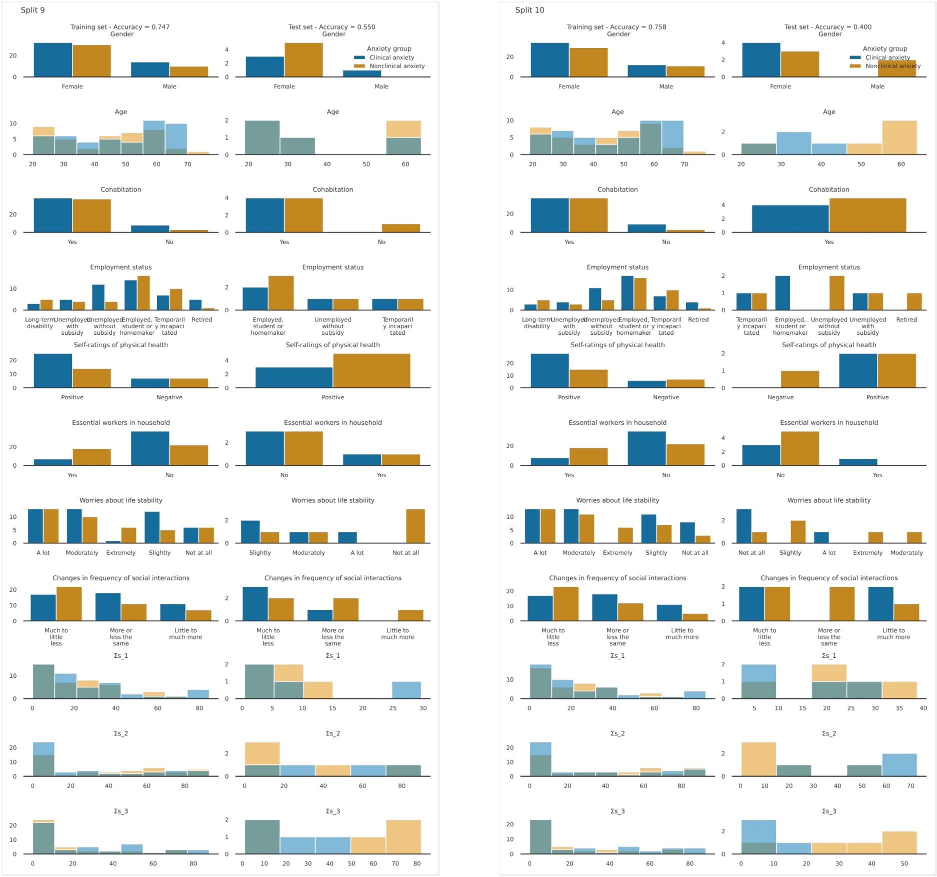

Table S5: Textual data encodings

Attribute	*Original value : Encoded Value*
Anxiety group based on the patients' GAD7 score	Nonclinical anxiety: 0; Clinical anxiety: 1
Self-ratings of physical health	NA: 0; Negative: 1; Positive: 2
Essential workers in household	NA: 0; No:1; Yes: 2
Worries about life instability	NA: 0; Not at all: 1; Slightly: 2; Moderately: 3; A lot: 4; Extremely:5
Changes in frequency of social interactions	Much to little less: 0; More or less the same: 1; Little to much more: 2
Employment status	NA: 0; Employed / homemaker / student: 1; Unemployed with benefits: 2; Unemployed without benefits: 3; Permanent disability: 4; Temporarily incapacitated: 5; Retired: 6
Cohabitation status	No: 0; Yes: 1
Gender	Female: 0; Male: 1

- NA = Not available
